# Use of Carnosine for Oxidative Stress Reduction in Different Pathologies

**DOI:** 10.1155/2016/2939087

**Published:** 2016-01-24

**Authors:** V. D. Prokopieva, E. G. Yarygina, N. A. Bokhan, S. A. Ivanova

**Affiliations:** ^1^Department of Biological Psychiatry and Narcology, Mental Health Research Institute, Tomsk 634014, Russia; ^2^Addictive States Department, Mental Health Research Institute, Tomsk 634014, Russia

## Abstract

The main properties and biological effects of the antioxidant carnosine, the natural dipeptide *β*-alanyl-L-histidine, are considered. Data on the effective use of carnosine in different pathologies are presented. Special attention is paid to issues of use of carnosine in neurologic and mental diseases, in alcoholism as well as in physiological states accompanied by activation of free-radical processes and formation of oxidative stress.

## 1. Oxidative Stress and Its Correction by Antioxidants

The pathogenesis of most diseases involves excess activation of free-radical processes and disturbance of functioning of the organism's antioxidant protection systems. This leads to increase in the level of reactive oxygen species (ROS) and forming of oxidative stress (OS). Mechanisms of OS formation in different pathologies are quite universal and are especially linked with disturbance of homeostasis and redox processes. The characterization of ROS, their types, main sources of formation in the organism, properties, and transformations are well described in various publications [1–3]. The main targets of damage under conditions of OS are proteins, lipids, carbohydrates, and nucleic acids.

It is well known that under normal physiological conditions ROS carry out important regulatory functions in the organism [1, 4]. However, under uncontrolled increase in ROS they interact with biomolecules, leading to their oxidative modifications. Products of such modification usually lose ability to carry out their functions. These products serve as “markers of oxidative stress,” and they include carbonylated, nitrosylated, and glycated proteins; aggregates due to crosslinking of protein molecules; products of lipid peroxidation (malondialdehyde, diene conjugates, hydroxynonenal, etc.); various types of hybrid adducts; advanced glycation end products (AGE products); dihydroguanosine, homocysteine, and so forth [1, 5]. All these products of oxidative damage of biomolecules are resistant to destructions and accumulate in cells, complicating their vital functions. Their neutralization can play an important role in correction of oxidative stress.

The search for and development of ways to correct oxidative stress is a relevant problem of modern medicine. One way that can be effective under clinical conditions involves the use of the so-called antioxidants, substances that neutralize ROS, reducing their reactivity in the organism. Despite the very large number of known antioxidants, to choose one for effective use in a specific clinical situation is quite difficult. This is caused by the abundance of factors of modification of macromolecules under OS. In addition, the mechanism of action of an antioxidant changes depending on its chemical structure, bioavailability, and damage rate of redox processes and severity of oxidative stress in the organism.

It was found that under conditions of oxidative stress, the endogenous systems of antioxidant response of the organism are activated through transcription factor Nrf2 [6]. Consequently, expression of endogenous antioxidant enzyme genes increased, increasing cellular defenses against detrimental redox modulations [7, 8].

The human endogenous antioxidant response system can regulate the amount of reactive species tightly and minimize related cellular damage. But the role of exogenous antioxidants is also important. It was found that exogenous antioxidants have a priming effect on the antioxidant response system [9]. Working together with the endogenous antioxidant response system, exogenous antioxidants allow for a more enhanced and efficient defense against detrimental redox modulations.

Extensive experimental and clinical material on the use of antioxidants has accumulated. In medicine, they are mainly used as additional agents to basic therapy. Many medications, in addition to their main therapeutic effect, also manifest antioxidant properties. However, depending on conditions and concentration, antioxidants can also show the opposite to antioxidant effect, that is, prooxidant action. Carotenes are polyunsaturated compounds; therefore, they can be oxidized via a radical mechanism and act as prooxidants [10]. Under certain conditions, for example, in the presence of mixed-valence metal ions, prooxidative effect is shown by ascorbate. Vitamin E as an antioxidant is most effective in a complex with other fat- and water-soluble reductants (ascorbic acid, ubiquinol, and flavonoids) in whose absence it is quickly inactivated or transforms into tocopheryl radical capable of initiating new chains of oxidation of unsaturated lipids; that is, it becomes a prooxidant as well [5, 11].

Adhering to the correct dosage of an antioxidant, as with every pharmacologically active compound, is very important. There are examples of ineffective use of antioxidants in the treatment of some pathologies accompanied by decrease in the level of antioxidants in blood plasma. So, clinical trials of the treatment of Alzheimer's disease with the addition of the well-known antioxidants lycopene and vitamins A, C, and E did not show positive results and even showed progressive decrease in cognitive function in the study participants in some cases [12]. Though these results do not favor antioxidant therapy, it might be due to prooxidative effects of these antioxidants under these conditions as well as terms and the scheme of their administration.

The choice of a specific antioxidant and exact indications and contraindications are still insufficiently developed for every specific disease. There is no information on the interaction of pharmaceuticals of natural origin with synthetic medications. In addition, antioxidants can cause allergic reactions, be toxic, and show low efficiency, and standardization is not always possible; the possibility of overdose also remains. Therefore, the search for substances with maximum antioxidant action and minimum side effects under conditions of OS continues and remains an important problem. Ideally, the antioxidant should show considerable antioxidant action within a broad range of concentration, be natural and hydrophilic, have good bioavailability, be nontoxic, not form toxic products during interaction with reactive oxygen species, not have negative effects in case of overdose, and have good compatibility with other medications.

In spite of the fact that the use of antioxidants in clinical practice does not always show positive results, the concept of use of antioxidant therapy is still relevant and has the potential for effective treatment of a number of disorders accounting for pathophysiological mechanisms of their forming and development.

## 2. Main Properties and Biological Effects of Carnosine

Numerous references as well as our own work experience indicate that the antioxidant carnosine, the natural dipeptide *β*-alanyl-L-histidine, meets almost all requirements for an ideal antioxidant. It is synthesized and contained in human muscle and nervous tissues, is easily absorbed in the digestive tract, penetrates through blood-brain barrier, and has high bioavailability and membrane-stabilizing action. Carnosine is a low molecular weight hydrophilic antioxidant of direct action, though it can also have an impact on the antiradical protection system of the organism [13]. Results of experiments on rats showed that carnosine accelerates the metabolizing of cortisol and noradrenaline released into blood of animals under stress, showing the mediation effect of carnosine [14]. Decrease in level of stress hormones in blood leads to a decrease in the severity of OS. In addition, carnosine is not addictive; there is no danger of overdose, and it does not accumulate in the organism during long-term administration because its surplus is cleaved by the enzyme carnosinase into amino acids that are easily eliminated from the organism [13]. However, it is noteworthy that there are cases of development of carnosinemia, a rare autosomal recessive [15] metabolic disorder [16] caused by a deficiency of carnosinase. This disorder results in an excess of carnosine in the urine, blood, and nervous tissue [17], and variety of neurological symptoms have been associated with carnosinemia [16, 18]; that is, under certain conditions, carnosine can exert negative effects.

There are publications in which positive biological effects of carnosine are explained by its pH-buffering properties [19]. However, carnosine is a buffer not only for protons, but also a buffer for mixed-valence metal ions and reactive oxygen species [20]. The ability of carnosine to form complexes with bivalent metals is known: with ions of copper, cobalt, manganese, and cadmium [21]. In another work, it was shown that carnosine binds iron ions [22]. Because ions of metals take an active part in many metabolic processes and can activate free-radical processes, the ability of carnosine to regulate the level of mixed-valence metal ions in the organism is one more important property of carnosine that confirms its antioxidant status.

Further, the antiglycating [23, 24] and the anticrosslinking [25] properties of carnosine have been shown, which are, in essence, reflections of its antioxidant effects, the ability to block oxidation of biomolecules.

Great contributions to the study of molecular mechanisms of protection of biomolecules by carnosine were made by Aldini et al. [26, 27]. Using liquid chromatography/electrospray ionization tandem mass spectrometry, they showed that carnosine and related peptides act as quenchers of reactive and cytotoxic carbonyl species through its ability to form adducts with them. This suggested that carnosine is a protector of biomolecules from oxidative/carbonyl stress. The ability of carnosine to react with carbonyls of proteins (termed “carnosinylation” of proteins) was reported by other authors [28], who considered this property of carnosine important for inactivation/removal of damaged proteins.

In culture of human cells, it has been shown that addition of carnosine into the medium at concentrations close to physiological (20–50 mM) increases longevity of the cells [29]. This was attributed to either reduction of the length of telomere fragments of chromosomes, lost by the cell during every doubling, or decrease in methylation of DNA. It could not be ruled out that carnosine decreases the accumulation of some other changes in DNA, whose accumulation above a critical point leads to termination of divisions.

It has also been reported that carnosine prevents toxic effects of hyperhomocysteinemia in rats [30]. It is known that homocysteine is a potent initiator of oxidative stress in many tissues. However, the molecular mechanism of such protection is not clear. Perhaps carnosine modulates affinity of glutamate receptors to homocysteine, prevents accumulation of ROS, or has other protective mechanisms. But it has been shown that these effects of carnosine are not connected with the improvement of homocysteine metabolism or the decrease in its concentration.

Data on the study of biological effects of carnosine shows that molecular mechanisms of its effects cannot always be explained only by antioxidant action. The exact molecular mechanisms of some effects of carnosine observed in experiment should be found. At the same time, obvious positive effect of this dipeptide allows using carnosine widely already in routine clinical practice.

Prospects for the use of carnosine in the treatment of some pathologies are reported in a report by Quinn et al. [31]. Data on the possible physiological role of carnosine based on its biochemical properties and study of the therapeutic potential of carnosine in a number of pathologies accompanied by oxidative or carbonyl stress are presented in a review by Boldyrev et al. [32].

## 3. Clinical Use of Carnosine

Researchers of the Kharkov Physiotherapeutic Institute were the creators of the first injection dosage form of carnosine. During subcutaneous injection of 0.5–1.0 mg, high therapeutic effectiveness in treatment of infectious and rheumatic polyarthritis and ulcer of the gastrointestinal tract was obtained [33]. Later, positive effect of carnosine in healing wounds of lung tissue was shown [34]. Japanese researchers played a great role in the study of a wound healing effect of carnosine. They created the agent Z-103 based on a complex of carnosine and zinc ions (L-carnosine-Zn^2+^) that has considerable antiulcer effect and reduces damage to the stomach lining induced by different forms of stress and chemical agents [35]. Japanese scientists also have priority for the use of carnosine in cancer diseases [36]. Carnosine combined with radiotherapy in treatment of patients with breast cancer considerably reduced side effects of radiation, injury of skin and intoxication of the organism, and increases immunity and increases likelihood of healing of treatment severalfold. Carnosine was effective also for the prevention of the cachexia caused by chemotherapy in cancer therapy [36]. In experimental studies on cultures of tumor cells, it has been shown that carnosine suppresses proliferation of human glioblastoma completely, and it decreases the level of reactive oxygen species and increases the activity of mitochondrial superoxide dismutase in tumor cells [37]. Possible mechanisms of inhibition of tumor cells growth by carnosine were considered recently [38].

The ability of carnosine to prevent age-related phacoscotasmus of the eye has been shown. Free-radical reactions leading to oxidative modification of lipids and proteins of crystallins of tissues of the eye are a basic reason for phacoscotasmus in senile cataract. In the development of cataract in the crystalline lens, a considerable decrease in the endogenous antioxidants glutathione and carnosine occurs. In clinical trials, efficiency of the agent in the form of eye drops for treatment of cataract containing 5% solution of carnosine has been shown. Later, when developing eye drops, a natural dipeptide, the relative of carnosine N-acetylcarnosine [13, 39], was successfully applied. Also, Chinese authors report the ability of carnosine to prevent development of cataract [40].

Carnosine in the form of 5% solution was also successfully used for the treatment of seasonal allergic rhinoconjunctivitis; thus, the need for additional administration of antihistaminic medications disappeared [13]. Carnosine found application also for the treatment of inflammatory diseases of parodentium for patients with fixed orthodontic designs: 5% solution of the dipeptide had a substantial immunocorrecting effect and increased activity of enzymes of antioxidant protection in saliva [41].

Carnosine was effective in treatment of diabetic complications in experimental studies on rats with streptozotocin-induced diabetes. It was found that treatment with carnosine (1 g/kg body weight per day) restored carnosine kidney levels, prevented podocyte loss, restrained glomerular apoptosis, and reduced expression of Bax and cytochrome C [42]. In rats with experimental diabetic retinopathy, carnosine exerted considerable protective effect on cells of capillaries of the retina [43]. Administration of carnosine (100 mg/kg injected daily) to mice with type 2 diabetes, to which experiment wounds were made (6 millimeters), enhanced significantly healing of wounds, which was accompanied by increased expression of growth factors and cytokine genes involved in wound healing [44].

Carnosine is applied successfully in cardiological practice. Addition of L-carnosine in cardioplegic solution during stopped heart operations allows increasing the operation duration severalfold without signs of necrotic damage of tissues of the heart in the operative field [45].

In experiments on rats with isoproterenol-induced myocardial infarction, it was shown that preliminary administration of carnosine (250 mg/kg/day i.p.) reduces cardiac toxicity of isoproterenol due to reduction of oxidative stress [46]. Use of carnosine in metabolic syndrome is promising, a state accompanied by oxidative stress and inflammation leading to development of diabetes and cardiovascular diseases [47]. There are also data indicating that carnosine has nephroprotective properties [48]. That report provides results of studies concerning the role of carnosine in kidney diseases, particularly in ischemia/reperfusion induced acute renal failure, diabetic nephropathy, gentamicin-induced nephrotoxicity, and blood pressure regulation.

Currently in Russia, a tableted dietary supplement under the name Sevitin is applied as a source of carnosine. It has been shown that this agent promotes recovery of cerebral circulation in chronic discirculatory encephalopathy and has a regulating effect on the activity of the immune system [49]. Studies are carried out focusing on obtaining new carnosine-containing agents for use under clinical conditions. There is a report on the creation and testing of nanocomplexes containing carnosine included in the structure of phospholipid nanostructures [50]. Use of such nanocomplexes provides resistance of carnosine to the action of carnosinase during its supply to the destination, which can significantly increase the influence of the dipeptide.

Recently, the question of reaching the effective concentrations of carnosine in tissues during its injection into the organism was specially studied on mice of C57 Black/6 line. It was shown that, after intraperitoneal administration of the agent at dose 1 g/kg, its maximum concentration in blood plasma is reached in 15 minutes. It was found that administration of exogenous carnosine could considerably increase its concentration in the brain: the maximum concentration of carnosine in the brain is reached 6 hours after injection, when the concentration of the agent in blood is the minimum [51].

## 4. Use of Carnosine in Neurologic and Mental Disorders

It is known that OS develops in Parkinson's and Alzheimer's diseases [52], acute ischemic stroke [53], schizophrenia [54], depression [55], addictive disorders, alcoholism [56–58], and so forth. Cells of the nervous system are very sensitive to free-radical oxidation due to many factors: high intensity of metabolic processes and high level of oxygen consumption; large amounts of lipids with polyunsaturated fatty acids; increased content of bound iron ions (oxidation inducers); low content of its transporters; formation of ROS during cellular metabolism of secondary messengers in neuronal cells; participation of free radicals in neuroregulation; low level of antioxidant protection in comparison with cells of other organs. This initiates an excitotoxic “chain reaction” in which neurons continually experience excessive extracellular glutamate levels and so forth [3, 5, 59].

This determines the special need for the protection of cells of nervous tissue against free-radical oxidation by natural antioxidants able to penetrate through the blood-brain barrier, such as carnosine.

Positive results were obtained during carnosine addition (2.0 g/day) to basic therapy of patients with chronic discirculatory encephalopathy. Such treatment led to increase in resistance of lipoproteins of blood plasma against Fe^2+^-induced oxidation, stabilization of erythrocytes against acid-induced hemolysis, intensification of respiratory burst of leukocytes, strengthening of endogenous antioxidant protection of the organism, and improvement of cognitive functions of the brain of patients [49], that is, carnosine exerted antioxidant, membrane-stabilizing, and immunomodulatory effects in this pathology.

Considerable improvement of clinical state of patients was observed during administration of carnosine at dose of 1.5 g/days for 30 days in addition to traditional therapy in treatment of Parkinson's disease [60]. Use of carnosine reduced toxic effects of basic therapy (side effects of antiparkinsonian agents). In patients, a statistically significant reduction of neurologic symptoms (improvement of coordination of movements) was observed. Positive correlation between activation of antioxidant enzyme of superoxide dismutase in erythrocytes and decrease in neurologic symptoms was revealed. Addition of carnosine in the scheme of treatment led to reliable decrease in hydroperoxides in lipoproteins of blood plasma and considerably increased the resistance low-density and very-low-density lipoproteins against Fe^2+^-induced oxidation and also reduction in amount of oxidized proteins in blood plasma. Thus, addition of carnosine to basic therapy not only improved considerably clinical indices, but also elevated the antioxidant status of the organism in patients with Parkinson's disease.

Carnosine was reported to have application also in schizophrenia. A randomized double-blind placebo-controlled study revealed that carnosine inclusion (2.0 g/days) as an addition to basic therapy in treatment of patients with schizophrenia improved their cognitive functions [61].

The protective activity of carnosine against zinc-induced neurotoxicity and its molecular mechanisms such as cellular Zn influx and Zn-induced gene expression were investigated using hypothalamic neurons (GT1-7 cells) [62]. The findings showed that carnosine could be effective in the treatment of vascular dementia, as Zn-induced neurotoxicity plays a crucial role in the pathogenesis of this disorder, and carnosine inhibits Zn-induced neuronal death.

Dietary supplementation with carnosine has been shown to suppress stress in animals and improve behaviour, cognition, and well-being in human subjects [63]. These results allow with great confidence assuming efficiency of treatment using carnosine for stress-related and depressive disorders.

## 5. Correction of Oxidative Stress with Carnosine in Alcoholic Patients

It has been reported that in alcoholic patients oxidative stress contributes strongly to forming somatic complications [64], disturbance of immune status [65], and induction of apoptosis [66]. In alcoholism, formation of OS can be increased by ethanol, the concentration of which significantly exceeds the norm in patients, as well as the toxic metabolite of ethanol-acetaldehyde, whose level also increases in the organism during alcoholic intoxication. Acetaldehyde can bind with many biological molecules (proteins of plasma, hemoglobin, factors of coagulant system of blood, lipids, etc.), forming with them aldehydic adducts that are deposited and accumulated in different tissues (liver, brain, heart, muscles, and intestines) [67, 68].

High indices of oxidative modification of biomolecules of membranes of erythrocytes and blood serum were found in alcoholic patients who were in the state of abstinence [69]. In other works, elevated content of carbonylated proteins and activity of aminotransferases of blood serum were revealed in patients with alcoholic delirium who were infected with hepatitis C or HIV virus [70]. The relationship between level of oxidation (carbonylation) of proteins of blood plasma and severity of manifestations of abstinence syndrome in patients was reported [71]. There is an opinion that a metabolic basis of developing alcoholic psychosis is the accumulation of acetaldehyde which, interacting with serotonin, forms toxic products having hallucinogenic properties [72]. It is known that, in patients with alcohol addiction, hyperhomocysteinemia is observed [73, 74]. The elevated concentrations of homocysteine stimulate entrance of Ca^2+^ and increase in ROS in the cytoplasm of neurons, which aggravates the state of OS. It has been reported that, in homocysteinemia, the functional activity of both nervous and immune systems of the organism decreases [30].

Thus, the activation of free-radical processes leading to accumulation of products of oxidative modification of biomolecules contributes considerably to the clinical course of alcoholism and can determine its features, which makes the study of effects of antioxidants in this pathology extremely important.

We have carried out several investigations on the effects of carnosine in alcoholism. In experiments* in vitro*, it is shown that addition of carnosine in tests with blood of alcoholics leads to increase in resistance of erythrocytes to acid hemolysis, promoting preservation of normal morphology of these cells [75].

A placebo-controlled study of the efficiency of carnosine in correction of OS in patients with alcohol addiction at the stage of forming of remission has been published [76]. Patients after basic treatment received carnosine at a dose of 1.2 g/day for one month before being released from the hospital. It was found that, after treatment in hospital, OS remained at a high level in patients. One month afterwards, during investigation in comparison group (patients who did not receive any agents at the stage of remission formation), severity of OS remained at the same level, as at baseline. In the patient group who received carnosine, reliable decrease in carbonylated proteins and products of lipid peroxidation (LP) in blood plasma to values corresponding to healthy persons was found. Intake of carnosine by patients for one month also led to an increase in the activity of superoxide dismutase of plasma and decrease in the activity of aminotransferases of blood serum. These results show that intake of carnosine effectively reduces severity of OS in the organism of alcoholic patients. Undesirable side effects were not observed. The mechanism of positive effect of carnosine on severity of OS in alcoholic patients remains unclear. However, our data on the ability of carnosine to prevent oxidative damage of proteins and lipids of blood induced by ethanol or acetaldehyde* in vitro* [77] show the ability of this dipeptide to protect biomolecules against direct toxic effects of ethanol and its metabolites.

## 6. Use of Carnosine in Physiological States Accompanied by Activation of Free-Radical Processes

Oxidative stress can develop not only in pathological processes, but also during considerable physical loads and during physiological aging of the organism. Therefore, carnosine now finds broad application as a general health-improving agent for healthy people under conditions of physical and psychological stress, during the impact of various adverse factors, and under extreme conditions. Carnosine is applied for the acceleration of recovery of tired muscles and increase in their working capacity in athletes [78] and in healthy elderly persons with active lifestyle [79]. Under experimental conditions, the geroprotective effect of carnosine has been shown. In experiments with the use of a specially bred line of rapidly aging mice, it was found that inclusion of carnosine in their diet leads to delay of aging of the animals due to increase in their antioxidant status [80].

The geroprotective effect of carnosine is mentioned in many publications where antioxidant, antiglycating, and anticrosslinking properties of carnosine are considered, because it was proven in the course of aging of the organism products of carbonylation, glycation, and cross-linking accumulate, which are well neutralized by carnosine.

Developments on the use of carnosine in the cosmetic industry are promising, which is confirmed by the available data on the ability of carnosine to prevent structural changes of collagen in skin and to prevent loss of its elasticity [81].

The cited data on successful use of carnosine in various pathologies and in physiological states accompanied by activation of free-radical oxidation shows prospects for its use as an effective antioxidant, a protector of tissues against various adverse factors inducing development of oxidative stress. Carnosine reduces action of factors whose excess in a cell has toxic effects.

## Abbreviations

ROS: Reactive oxygen species
OS: Oxidative stress
AGE products: Advanced glycation end products
Nrf2: Nuclear factor (erythroid-derived 2) like 2.
